# Buffalo Medical and Surgical Journal, Vol. xviii

**Published:** 1878-08

**Authors:** 


### Editorial.

### Buffalo Medical and Surgical Journal, Vol. xviii.

It is now seventeen years since we commenced our first efforts to furnish the profession of Buffalo and vicinity a medium of communication with the medical public, and with the present number we reach Vol. XVIII. Imperfections and failures have given variety to our more general successes, teaching us how we depend upon the profession for the reputation, interest, value and continuance of the Journal. If the physicians of Buffalo and vicinity take interest in and support the Journal by their contributions and subscriptions, their influence, example and money, the success of the enterprise is assured. By their aid the editor has been able thus far to conduct their organ, and from long association has acquired a personal interest in its success and continuance, and often made sacrifices of time and capital to insure its permanency. He has at times grown weary of the labor and tried to transfer it to more active hands, but the depression in general business has affected the financial receipts of the Journal to such degree that young and new-made fortunes were inadequate to pay the publication or furnish cash for the unpaid subscriptions, now, some of them many years in arrears. These appear small, singly, but in the aggregate amount to several thousand dollars, and attention is called to this fact in the trust that our friends and subscribers will realize the necessity to the Journal, of new subscribers and "*pay in advance.*" It will thus be seen that the past seventeen years of our Journal has been attended by anxieties and embarrassments, though yet always sustained and comforted by a large number of faithful and tried friends whose unfaltering support has been an unfailing trust.

Thus much for our past history; though it contains no mention of achievements and other compensations, while we leave each reader to judge for himself of our real success, after he has considered the medical atmosphere, where our Journal has been obliged to breath.

Vol. XVIII commences its life at a time of great activity, never perhaps in the history of our profession has medicine and surgery been undergoing more rapid or more progressive change. Great principles are being settled, practical questions in constant application are now preeminently considered, and the "millenium" of our profession seems just at hand. We shall doubtless be able to chronicle its full establishment in the new volume. Our readers are invited to aid in the great work and send in copy at an early date.

